# Anthropogenic N input increases global warming potential by awakening the “sleeping” ancient C in deep critical zones

**DOI:** 10.1126/sciadv.add0041

**Published:** 2023-02-08

**Authors:** Shuping Qin, Haijing Yuan, Chunsheng Hu, Xiaoxin Li, Yuying Wang, Yuming Zhang, Wenxu Dong, Timothy Clough, Jiafa Luo, Shungui Zhou, Nicole Wrage-Mönnig, Lin Ma, Oene Oenema

**Affiliations:** ^1^ Hebei Provincial Key Laboratory of Soil Ecology, Key Laboratory of Agricultural Water Resources, Center for Agricultural Resources Research, Institute of Genetic and Developmental Biology, The Chinese Academy of Sciences, 286 Huaizhong Road, Shijiazhuang 050021, Hebei, China.; ^2^ Department of Agriculture and Life Sciences, Lincoln University, Lincoln, New Zealand.; ^3^ Land and Environment, AgResearch, Hamilton 3240, New Zealand.; ^4^ Fujian Provincial Key Laboratory of Soil Environmental Health and Regulation, College of Resources and Environment, Fujian Agriculture and Forestry University, Fuzhou 350002, China.; ^5^ Department of Agriculture and the Environment, Grassland, and Fodder Sciences, University of Rostock, Rostock, Germany.; ^6^ Wageningen University and Research, Wageningen, Netherlands.

## Abstract

Even a small net increase in soil organic carbon (SOC) mineralization will cause a substantial increase in the atmospheric CO_2_ concentration. It is widely recognized that the SOC mineralization within deep critical zones (2 to 12 m depth) is slower and much less influenced by anthropogenic disturbance when compared to that of surface soil. Here, we showed that 20 years of nitrogen (N) fertilization enriched a deep critical zone with nitrate, almost doubling the SOC mineralization rate. This result was supported by corresponding increases in the expressions of functional genes typical of recalcitrant SOC degradation and enzyme activities. The CO_2_ released and the SOC had a similar ^14^C age (6000 to 10,000 years before the present). Our results indicate that N fertilization of crops may enhance CO_2_ emissions from deep critical zones to the atmosphere through a previously disregarded mechanism. This provides another reason for markedly improving N management in fertilized agricultural soils.

## Teaser

Agricultural N fertilization is triggering the release of thousands-years-old SOC a dozen meters underground.

## INTRODUCTION

Globally, the top 2-m soil layer contains ~3000 Pg of soil organic carbon (SOC), which is threefold larger than the 770 Pg of CO_2_-C currently in the atmosphere (*1*). In addition, the soil layers deeper than 2 m, together with the friable weakly weathered bedrock (defined here as deep critical zones), represent another large SOC stock (*2*). Land use commonly determines whether the topsoil is a source or sink of atmospheric CO_2_ (*3*–*5*), but land use is thought to have little influence on SOC in deep critical zones (*6*–*8*). Thus, the stability and residence time of SOC increases strongly with increasing soil depth (*4*, *8*–*10*). The SOC in deep critical zones is much less susceptible to anthropogenic impacts than that in the surface soil because of the buffering provided by the upper soil layers (*6*, *11*).

Anthropogenic fixation of dinitrogen (N_2_) began over a century ago, resulting in the generation of reactive N species that enhance soil fertility and crop production. Now, the global anthropogenic N input (~210 Tg N year^−1^ in 2010) exceeds all the natural processes combined (*12*, *13*). A considerable fraction of the anthropogenically fixed N (600 to 1800 Tg) has been stored in deep critical zones as nitrate (*14*), especially in well-drained and intensively fertilized areas (*15*, *16*). So far, little is known about whether and how this increase in reactive N affects the SOC stability in deep critical zones.

Most studies examining the effects of N addition on SOC mineralization have been limited to the topsoil or, at most, the surface 0 to 1 m depth, with positive, negative, or no effects of N additions observed on SOC mineralization in surface soils (*17*–*20*). This inconsistency in reported results is likely to be caused by two counteracting mechanisms: positive effects of N addition on the decomposition of labile SOC versus negative effects of N addition on the decomposition of mineral-associated SOC (*20*). Unlike the surface soil, the deep critical zones have much lower rates of new C inputs [e.g., turnover of plant roots, burrowing fauna, bioturbation, and dissolved organic carbon (DOC) leaching] (*6*, *8*). In addition, oxygen (O_2_), as the preferred electron acceptor for SOC oxidation, is also in short supply in deep critical zones (*21*). Thus, microbes may lack energy to decompose, and lack O_2_ to oxidize, the SOC within deep critical zones (*8*, *9*). Consequently, the facultative microbes, which respire nitrate instead of O_2_, may influence the biogeochemical cycling of SOC in deep critical zones.

## RESULTS AND DISCUSSION

**Fig. 1.**
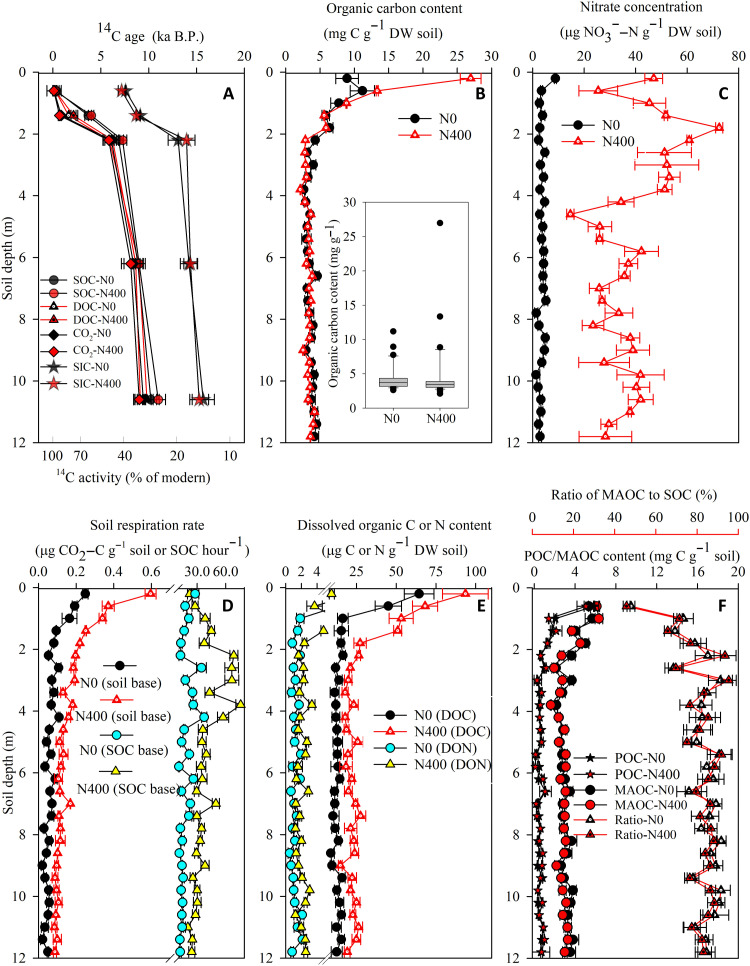
Vertical distributions of soil C and N contents and mineralization through the 0- to 12-m soil profile under N0 and N400 treatments. (**A**) ^14^C activities and ^14^C ages of SOC, DOC, and CO_2_. (**B**) SOC contents. (**C**) Nitrate contents. (**D**) Soil respiration rates. (**E**) DOC and DON contents. (**F**) Particulate organic carbon (POC) and mineral-associated organic carbon (MAOC) contents. DW, dry weight; DON, dissolved organic nitrogen; N0, soil with nil fertilizer N input since 1998; N400, soil with a fertilizer N input of 400 kg N ha^−1^ year^−1^ since 1998. The inlay in (B) shows the boxplot of profile SOC contents. Values are given as means ± SEM (*n* = 3).

This study investigated whether, and how, long-term anthropogenic N inputs might affect SOC mineralization in the deep critical zone (between 2 and 12 m), in soil incubations of a long-term, controlled, N fertilization experiment located in the Luancheng experimental station of the Chinese Academy of Sciences (37.90°N, 114.70°E; elevation of 50 m) in the North China Plain. The soil is a Haplic Cambisol developed on deep alluvial loess deposits with a silt loam texture. Globally, Cambisols cover 10% of the terrestrial surface and are the dominant cultivated soil types (*22*). Thus, results acquired using this Cambisol soil, examining the effects of nitrate loading on deep SOC mineralization, are widely applicable. Radiocarbon (^14^C) dating showed that the mean age of the SOC increased sharply from 0.4 to 6.9 thousand years (ka) before the present (B.P.) over the 0- to 2-m soil depth and then increased slowly from 6.9 to 11.0 ka B.P. over the 2- to 12-m soil layers (Fig. 1). These results indicate that most of SOC in the 2- to 12-m depth is ancient organic C. While the SOC concentration declined with depth, the 2- to 12-m-deep soil still accounted for as much as 30% of the total SOC stock in the 0- to 12-m soil profile (Fig. 1).

The area of the study site was previously native grassland (mainly *Artemisia*, *Chenopodiaceae*, *Poaceae*, and *Xanthium*) before being used for wheat production over 1 ka ago (*23*). A winter-wheat, summer-maize, double cropping system (two crops per year) has been practiced since the 1970s. A long-term field experiment with different N fertilization rates has been ongoing since 1998. Before 1998, the N fertilization rate of the experimental site was <100 kg N ha^−1^ year^−1^ with no obvious nitrate accumulation observed in the soil profile. Compared with the nil N fertilizer control (N0 treatment), a 20-year N input of 400 kg N ha^−1^ year^−1^ (N400 treatment) has increased the nitrate concentration, on average, 4.6-fold over the 2- to 12-m soil depth (Fig. 1). Excess N fertilization and limited irrigation/precipitation are prerequisites for nitrate accumulation in the deep critical zones (*24*–*27*). Competitive adsorption with other anions (e.g., chloride, sulfate, and carbonate) is considered to be a dominant mechanism for retaining nitrate within the deep critical zones (*28*, *29*).

The soil respiration rates in the 2- to 12-m soil profile were, on average, 92% higher under the N400 treatment than under the N0 treatment (Fig. 1). The in situ temperature in the upper 2- to 3-m layer of deep critical zone at the experimental site ranges from 8° to 20°C (*30*). With increasing soil depth, the soil temperature is expected to have smaller seasonal variation and finally approach the annual mean air temperature of the experimental site (12°C) (*30*). To test whether the ambient temperature affected the promotion of soil respiration under elevated nitrate, we incubated the deep soil samples from both the N0 and N400 treatments under temperatures ranging from 8° to 20°C. The results showed that nitrate addition significantly stimulated deep soil respiration regardless of the ambient temperature (fig. S1). Both the ^14^C age and the ^13^C isotopic signature (δ^13^C) of the CO_2_ emitted during the incubation of the deep critical zone soil were consistent with those of the SOC but differed from those of the inorganic C (Fig. 1 and fig. S2). These results indicate that the CO_2_ being emitted during the incubation of the deep soil was derived from the ancient SOC, rather than from inorganic C. Our results suggest that 20 years of constant N inputs can accelerate the mineralization of ancient SOC in the deep critical zone up to 12 m depth.

**Fig. 2.**
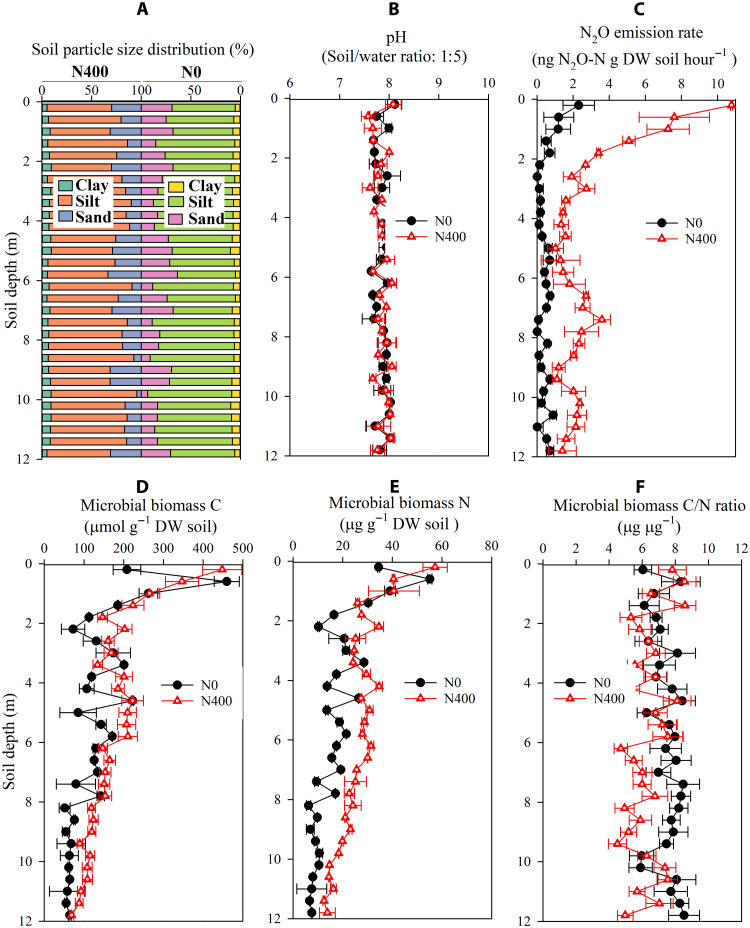
Vertical distributions of soil texture, pH, N_2_O emission, and microbial biomass through the 0- to 12-m soil profile under N0 and N400 treatments. (**A**) Soil mineral particle size distribution. (**B**) pH value. (**C**) N_2_O emission rate. (**D**) Microbial biomass C content. (**E**) Microbial biomass N content. (**F**) Microbial biomass C/N ratio. Values are given as means ± SEM (*n* = 3).

The stabilization of SOC in the deep critical zone has been increasingly attributed to the distinctive environmental conditions within this zone (*4*, *9*). First, the limited availability of O_2_, as the preferred electron acceptor for microbes to oxidize SOC, could retard SOC oxidation in deep soil. Nitrate, as a competing electron acceptor, relative to O_2_, can be used for microbial respiration in conditions where the molar concentration ratio of nitrate to dissolved O_2_ exceeds 3.8:1 (*31*). In this study, the accumulated nitrate could have acted as an alternative electron acceptor for microbial respiration in the deep critical zone because the molar concentration ratio of nitrate to dissolved O_2_ was much higher than 3.8:1 (fig. S3). In support, the N_2_O emission rate from deep soil samples during the incubation was, on average, 4.8-fold higher under the N400 treatment than under the N0 treatment (Fig. 2). To test whether nitrate acted as an alternative electron acceptor to O_2_, we investigated the effects of nitrate addition on deep soil respiration at this site, and a second soil from the loess plateau, under different O_2_ concentration conditions. The results show that O_2_ level determined whether nitrate addition stimulated deep soil respiration (fig. S4). Nitrate addition significantly increased the respiration rates in both deep soils when the soil air O_2_ concentration was below 5% (fig. S4). This result demonstrates that the promoting effects of nitrate on deep soil respiration, which results from the role of nitrate as an alternative electron acceptor to O_2_, can be extrapolated to other areas where deep critical zones occur.

**Fig. 3.**
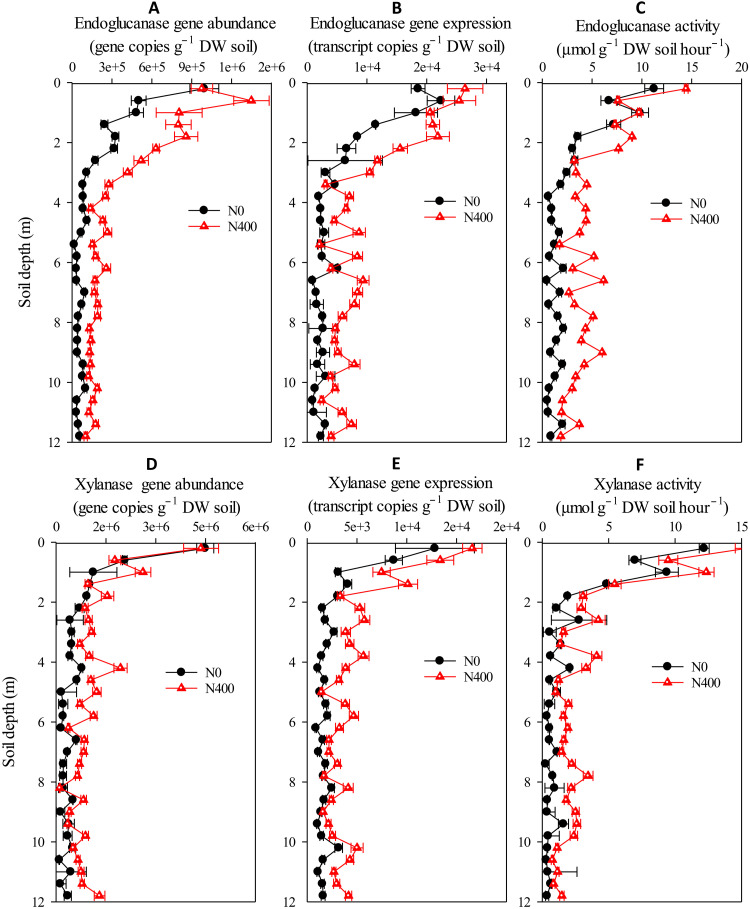
Vertical distributions of typical SOC-degrading gene abundances and expressions and enzyme activities through the 0- to 12-m soil profile under N0 and N400 treatments. (**A**) Endoglucanase gene abundance. (**B**) Endoglucanase gene expression. (**C**) Endoglucanase activity. (**D**) Xylanase gene abundance. (**E**) Xylanase gene expression. (**F**) Xylanase activity. Values are given as means ± SEM (*n* = 3).

Other widely recognized mechanisms for stabilizing the SOC in the deep critical zones include chemical association of SOC with mineral surfaces and the physical occlusion of SOC within mineral structures (*32*). In this study, the mineral-associated organic carbon (MAOC) accounted for 80 to 90% of the SOC in deep critical zones (Fig. 1F), indicating that most of the SOC there was chemically bound with mineral surfaces or/and physically occluded within mineral structures, which decreased its accessibility to decomposers (*33*). The accumulated nitrate in the deep critical zone under the N400 treatment was a nutrient (N) for microbial reproduction, thereby causing significant increases (*P* < 0.01, Student’s *t* test) in the microbial biomass, functional gene expression, and enzyme activities relevant to SOC decomposition under the N400 treatment compared to the N0 treatment (Fig. 3). Thus, the enhanced microbial biomass of the SOC decomposers and their associated enzymes increased the probability of their interactions with the mineral-associated SOC, which accounts for the promotional effect of nitrate on SOC decomposition in the deep critical zones.

The DOC concentrations were, on average, 57% larger under the N400 treatment compared with the N0 treatment (Fig. 1). The maximum rooting depth previously reported for winter wheat was 2.0 m and for maize was 1.2 m at this site (*34*). The ^14^C dating indicated that the DOC in the deep critical zone had a similar age to the ancient SOC within this zone (Fig. 1). These results indicated that the increased DOC in the deep critical zone under the N400 treatment was unlikely derived from crop roots but from the decomposition of the ancient SOC. The increase in DOC could have been a consequence of, or a reason for, the increase in microbial activity under the N400 treatment when compared with the N0 treatment. To clarify this ambiguity, we investigated the dynamics of both the microbial biomass and the DOC concentrations at the current study site and the soil from the loess plateau, after nitrate amendment. The results showed that, during the first week of the incubation, nitrate addition significantly increased microbial biomass, but did not affect DOC concentration (fig. S5). After 2 weeks of incubation, the DOC concentrations differed significantly between the nitrate addition treatment and the control treatment (fig. S5). These results demonstrated that nitrate first promoted deep soil microbial proliferation. This was then followed by enhanced microbial activity that, in turn, decomposed the mineral-associated SOC to release DOC. Last, a positive feedback occurred between the DOC release and microbial activities (*9*, *35*), which accounted for the observed increases in both the DOC concentration and soil respiration within the deep critical zones after long-term N fertilization (Fig. 1).

The present study shows that the anthropogenic N inputs from excessive N fertilization can “waken” the “sleeping” ancient buried SOC and liberate CO_2_ from the deep critical zone. The SOC concentrations in the deep critical zone tended to be slightly smaller under the N400 treatment than under the N0 treatment; however, the difference was not statistically significant (*P* > 0.05, Student’s *t* test) in most of the soil layers (Fig. 1). This lack of statistical difference is likely due to the fact that the SOC stock and its spatial heterogeneity were relatively large when compared with the respiration flux. We postulate that an even longer-term experiment would be needed to be able to observe significant differences in SOC stock under different N inputs.

Our results imply that aboveground anthropogenic N inputs can release the ancient sequestered SOC deep within the critical zone. Increasing N inputs from atmospheric N deposition and N fertilization increase the risk of nitrate leaching and can stimulate the loss of ancient buried SOC in deep critical zones. Our results can be generalized to many semiarid areas with thick vadose zones, where the nitrate stocks have been substantially increased because of anthropogenic N inputs over recent decades (*14*, *36*). Globally, the annual soil respiration is estimated to be approximately 100 Pg C year^−1^ (*37*). The contribution of deep critical zones to global soil respiration is still poorly understood. Given that 85% of the global land is estimated to have critical zones with thicknesses deeper than 2 m (*38*), and that N inputs have been increasing during recent decades, there is a risk that the ancient SOC in deep critical zones is potentially being lost on large areas of land. Forecasts indicate that N inputs to agricultural land will increase further during the next few decades (*39*). Our study highlights a previously disregarded interaction between N fertilization and CO_2_ emissions from well-drained deep critical zones and provides another reason for the need to markedly improve N management in agriculture and to minimize residual nitrate in soil. Improved N management will reduce not only N_2_O emissions from surface soils (*40*) but also CO_2_ and N_2_O emissions from well-drained agricultural lands with deep critical zones.

## MATERIALS AND METHODS

### Experimental site and soil

A long-term N fertilization field experiment has been conducted since 1998 at the Luancheng Agro-Ecosystem Experimental Station (37°90′N, 114°70′E) in the North China Plain. Two N fertilization levels have been applied: 0 and 400 kg N ha^−1^ year^−1^, labeled N0 and N400, respectively. Each treatment had three replicates. The plot size was 7.0 m × 10.0 m. The 0- to 12-m soil profile has a silt loam texture and is classified as a Haplic Cambisol (Fig. 2A). The crop regime is a summer-maize winter-wheat rotation (two crops per year), which is the predominant crop system in the North China Plain. Information on the climate of the study area, the fertilizer, water, and other field managements have been reported previously (*30*).

### Soil sampling and stratifying

Intact soil columns from the soil surface to 12 m depth were collected from the experimental plots (one soil column for each plot), using a vehicle-mounted Geoprobe drilling rig (Geoprobe 54DT, USA). The rig has a dual tube system with a plastic, polyvinylchloride transparent liner (1.2 m long and 43 mm inner diameter) within a hollow steel rod. The rod was pushed to the required depth by percussive and static force with the intact soil cores piled into the liner. Extreme care was taken to prevent contamination of subsoil samples by topsoil samples. The soil columns within the liners were then immediately transported into an anaerobic glove box and divided into 30 sections (0.4 m per section), with both ends of each liner sealed with plastic films to prevent water loss and O_2_ exposure before measurements. The CO_2_ and N_2_O emission rates of each section of the intact field-moist soil columns were measured using a robotic system as explained below. After CO_2_ and N_2_O emission determinations, the soil from each layer was anaerobically homogenized and divided into two subsamples. One subsample was stored in a plastic bag at 4°C until soil physicochemical and respiration rate analyses were carried out. The other subsample was stored in a plastic bag at −80°C for soil microbial community structure, functional gene abundance, and expression and enzyme activity assays.

### Soil respiration and N_2_O emission assays

Soil respiration rate was determined by quantifying CO_2_ emitted from the intact field-moist soil columns. The main procedure was as follows: Each section of the intact field-moist soil column was removed from the liner and then placed in a 1016.5-cm^3^ cylindrical container (4.3 cm in inner diameter and 70.0 cm in height). The volume of the soil core and the headspace was 580.9 and 435.7 cm^3^, respectively. A small removable electric fan with battery was placed in the upper edge of the container to mix the gases in the headspace. The containers were capped with an air-tight butyl rubber septum and a plastic cap with thread. All of the above operations were conducted in a glove box to exclude the soil column being exposed to the ambient air concentration of O_2_. For the soil columns in the top 0- to 2-m layers, the O_2_ concentrations of the headspace gases in the containers were adjusted to the in situ soil-air O_2_ concentrations (the details for in situ soil-air O_2_ concentration measurements are described in Supplementary Methods; fig. S6). For the soil columns deeper than 2 m, the O_2_ concentrations of the headspace gases in the containers were adjusted to 5% (v/v) because the in situ soil-air O_2_ contents, in the soil deeper than 2 m, were consistently at this concentration (fig. S6). After the containers were sealed, they were incubated in the dark in a thermostatic water bath at 20°C for 30 hours. The headspace gases within the containers were sampled every 6 hours (2.0 ml per sampling) and analyzed using a robotized incubation and analysis system that has previously been described in detail (*41*). Briefly, the system has three main components: a thermostatic water bath, an autosampler (Gilson Model 222, Gilson, France), and a gas chromatograph (GC; Agilent, 7890A) connected with a peristaltic pump (Gilson Miniplus 3). The GC analyzed the concentration of N_2_O and CO_2_ using an electron capture detector and thermal conductivity detector, respectively (*41*). Changes in CO_2_ and N_2_O concentrations over time were used to calculate the CO_2_ and N_2_O emission rates by linear regression.

### Soil basic physicochemical parameter assays

Soil texture was determined using a laser particle analyzer (Malvern Mastersizer 3000, UK) (*42*). Gravimetric soil water content was determined after oven-drying (105° ± 0.5°C for 24 hours) a subsample. Soil nitrate concentration was measured by dual-wavelength ultraviolet spectrophotometry [after extraction with 1 M KCl solution; extraction ratio, 1:5 (w/v)] (*43*). Soil DOC and DON (dissolved organic nitrogen) concentrations were determined after extracting field-moist soil with distilled water [1:5 (w/v)] using a TOC analyzer (Elementar liquid TOCII, Germany) (*44*). After DOC concentration determination, the remaining soil DOC extracts were freeze-dried to concentrate the DOC for ^14^C dating measurements (see below). The total SOC content was measured using a dichromate oxidation method (*45*). The particulate organic carbon (POC) and MAOC were separated by the wet sieving method (*46*). Briefly, soil samples were dispersed by shaking in 0.5% sodium hexametaphosphate solution for 18 hours and then rinsed onto a 53-μm sieve. The fraction remaining on the sieve was POC, while the fraction passing through the sieve was MAOC (*46*). The carbon content of each fraction was assayed using an elemental analyzer (LECO TruSpec CN). Soil pH was determined in a 1:5 suspension of soil in distilled water using a glass electrode (*47*).

### SOC degradation genes and enzyme activity assays

Total DNA and RNA were extracted using the E.Z.N.A. Soil DNA and RNA Kit (Omega Biotek 456 Inc., Norcross, GA), respectively, according to the manufacturer’s instructions. The extracted DNA and RNA were used to measure the SOC degradation genes’ abundance and expression after checking the quality and quantity of the extracted DNA or RNA using a NanoDrop spectrophotometer (NanoDrop ND-2000c Technologies Inc., Wilmington, DE). The extracted RNA was used to synthesize cDNA with random primers and SuperScript III reverse transcriptase (Invitrogen). Two typical genes involved in recalcitrant SOC degradation, endoglucanase (EC 3.2.1.6) and xylanase (EC 3.2.1.8), were quantified using quantitative polymerase chain reaction (PCR). The primers, reagents, and cycling conditions were as described previously (*48*, *49*). The potential activities of soil endoglucanase (EC 3.2.1.6) and xylanase (EC 3.2.1.8) were measured using the dinitrosalicylic acid method (*50*).

### ^13^C and ^14^C abundances of SIC, SOC, DOC, and CO_2_

Before determining the ^13^C and ^14^C abundances, the species of soil inorganic carbon (SIC), SOC, and DOC were transformed into CO_2_. First, the soil samples were air-dried and sieved through a 1-mm mesh sieve to remove the gravels. Excessive dilute HCl (concentration, 10%; HCl solution/soil ratio, 2:1; sufficiency of HCl amount verified by repeated HCl addition to test samples) was added to the soil samples to completely transform the SIC species (e.g., inorganic carbonates) into CO_2_, which was collected for the determination of ^13^C and ^14^C abundances of SIC. Then, the dilute HCl-treated soil samples were repeatedly washed with pure water until the washing solution became neutral. The washed soil samples and the prepared soil extracts were freeze-dried and combusted to transform the SOC and the DOC into CO_2_, respectively (*9*, *51*). Last, the produced CO_2_ was collected to determine the ^13^C and ^14^C abundances of SOC and DOC.

The natural abundances of ^13^C of the prepared CO_2_ samples were determined using a continuous flow isotope ratio mass spectrometer and presented in standard notation (*51*). The ^14^C activities were determined using the liquid scintillation counting method. First, the prepared CO_2_ samples were purified using dry ice and liquid nitrogen trap and then converted to benzene under catalysis (*52*). Then, the synthesized benzene samples were left for 4 weeks to allow radon decay (*52*). Last, the benzene samples were mixed with a scintillation cocktail, and the ^14^C activity of the synthesized benzene was assayed using an ultralow-level liquid scintillation spectrometer (Quantulus, 1220) with a counting time of 20 hours (*53*). The ^14^C ages of SIC, SOC, DOC, and CO_2_ were calculated using the Libby half-life (5568 years) and corrected for isotopic fractionation according to methods published previously (*9*). The ^14^C ages are expressed as years before the present.
