# Supplementary material for: Anthropogenic N input increases global warming potential by awakening the “sleeping” ancient C in deep critical zones
Source: Sci Adv. 2023 Feb 8;9(6):eadd0041. doi: 10.1126/sciadv.add0041 (PMC9908017; doi:10.1126/sciadv.add0041)
Supplement: Supplementary file 1 — Supplementary Methods Figs. S1 to S6 [file sciadv.add0041_sm.pdf]

Science Advances
AAAS

Supplementary Materials for

Anthropogenic N input increases global warming potential by awakening the "sleeping" ancient C in deep critical zones

Shuping Qin *et al.*

Corresponding author: Chunsheng Hu, cshu@sjziam.ac.cn

Sci. Adv. **9**, eadd0041 (2023)
DOI: 10.1126/sciadv.add0041

This PDF file includes:

Supplementary Methods
Figs. S1 to S6

Supplemental Information

1. Supplemental methods

1.1 Soil air O_2 concentration measurement

The soil air in the 25–30, 55–60, 85–90, 145–150, 195–200, 245–250 and 295–300 cm depths was collected using soil–air equilibration samplers which were installed in each field plot of the long-term N fertilization experiment. Briefly, the samplers consisted of soil–air equilibration chambers pre-installed in the different soil layers with microbore polytetrafluoroethylene tubing connecting with three-way stopcocks at the soil surface. The details of the construction and installation of the soil–air equilibration sampler have been previously reported(*30*). Soil air samples for each layer were collected using 100 ml plastic syringes that were connected with the three-way stopcock of the sampler on the soil surface. Before sampling, a volume of gas (equal to the internal volume of the connecting tubing for the corresponding soil layer) was extracted to exclude the gas within the tubes. The O_2 concentrations in the gas samples were determined by gas chromatography (GC, Agilent 7890A) with a thermal conductivity detector.

1.2 Effects of incubation temperature on deep soil respiration under N0 and N400 treatments

In order to test whether the promoting effect of the added nitrate on deep soil respiration was dependent on ambient temperature, the soil samples of the 9.6-10.0 m sections under the N0 and N400 treatments were incubated with temperatures that ranged from 8-20 ºC for 5 days. The CO_2 emissions were measured using a gas chromatograph equipped with a thermal conductivity detector as described in the Method sections of the main text.

1.3 Effects of soil-air O_2 levels on deep soil respiration with and without nitrate addition

In order to test whether the stimulating effects of nitrate on deep soil respiration is caused by its role as an alternative electron acceptor to O_2, the cambisol soil samples from the 9.6-10.0 m depth under the N0 treatment and a loess soil sample of 9.6-10.0 m sections from the Suide county of Shaanxi Province, China, were taken. The nitrate and SOC concentrations of the loess soil sample were 3.6 mg N kg^{-1} and 1.2 g C kg^{-1}, respectively. The soil sampling procedure was described in detail in the Methods section. Then 30.0 g field-moist soil samples were incubated with an ambient O_2 level of either 0, 3, 5 and 8 %. Two treatments, a nitrate addition (50 mg NO_3^--N kg^{-1} dried soil) or a control treatment, were conducted for each O_2 level (n=3). The procedures for the nitrate addition and O_2 level adjustment were identical to the procedures described in the Methods section. The soil samples were incubated under the given O_2 levels at 12 ºC for 5 days and the CO_2 emissions were measured as described above.

1.4 Effects of nitrate addition on the dynamics of deep soil DOC concentrations and microbial biomass

In order to test whether the increased DOC concentration fostered microbial reproduction, or vice versa, 30.0 g of the 9.6-10.0 m depth cambisol soil under the N0 treatment and from the loess soil were incubated at an O_2 level of 5 % and a nitrate addition of 50 mg NO_3^--N kg^{-1} dried soil. The soil samples were incubated under 12 ºC for 30 days. Thirty replicates for each soil type were conducted. Three replicates were randomly selected for each soil on the 1st, 2nd, 3rd, 5th, 8th, 12th, 17th, 24th and 30th day of the incubation, respectively. The DOC and microbial biomass C and N contents were measured using the procedures described in the Methods section.

2. Supplemental figures

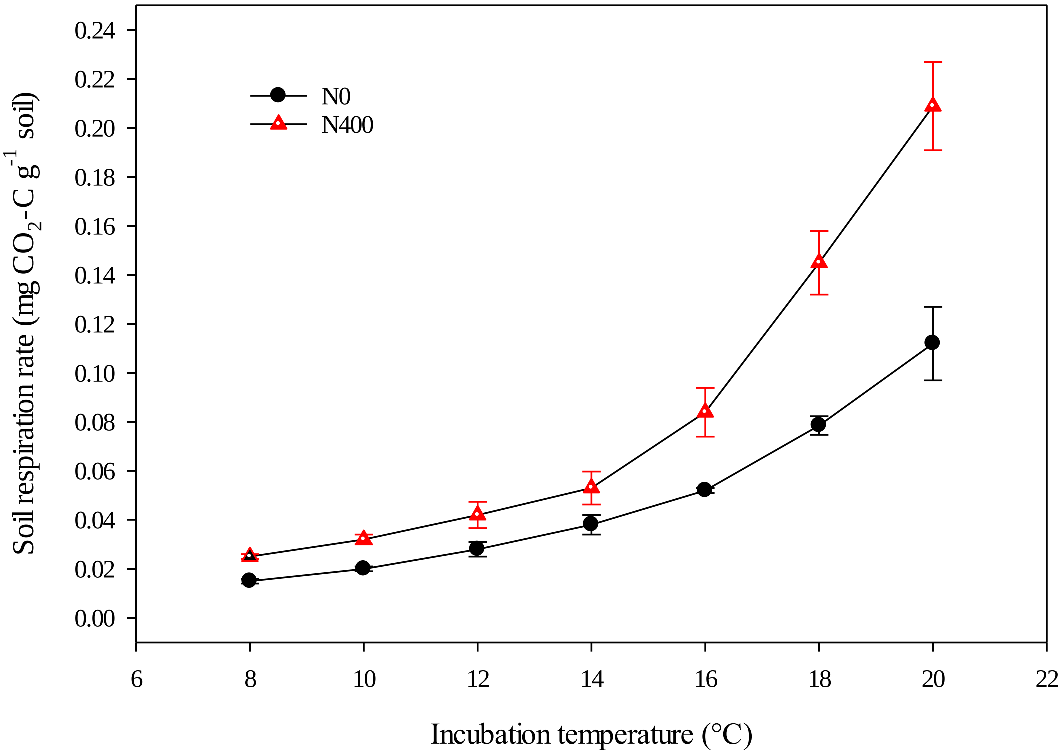

Fig. S1 Effects of incubation temperature on soil respiration for soil from the 9.6-10.0 m depth under the N0 and N400 treatments. Values are given as mean ± SEM (n=3).

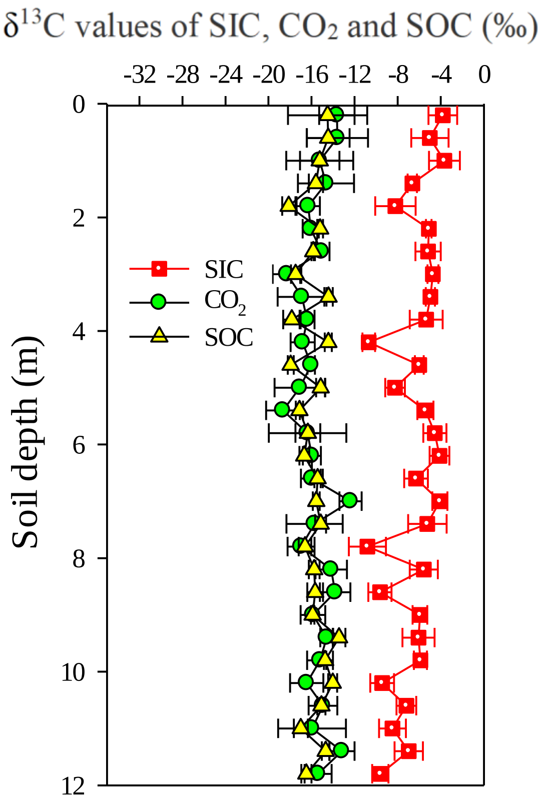

Fig. S2 Vertical distributions of the δ ^{13}C values of soil inorganic carbon (SIC), soil organic carbon (SOC) and the CO_2 emitted during the incubation. Values are given as mean ± SEM (n=3). The soil samples were collected from the field plots of the N400 treatment.

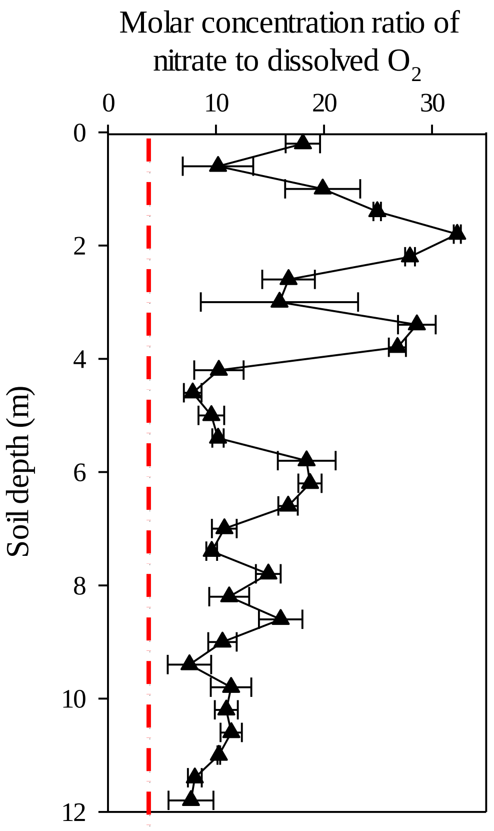

Fig. S3 Vertical distributions of the molar concentration ratio of nitrate to dissolved O_2 in soil solution through the 0-12 soil profile under a fertilizer-N input of 400 kg N ha^{-1} yr^{-1} since 1998. The red dotted line represents the threshold value of 3.8 above which microbes can use nitrate as a competing electron acceptor versus O_2. Values are given as mean ± SEM (n=3).

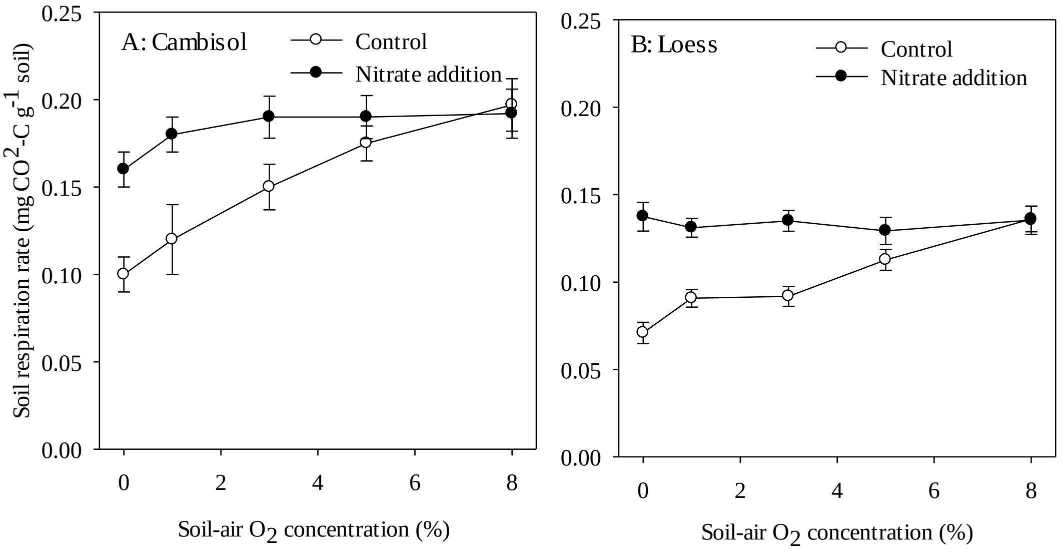

Fig. S4 Effects of soil-air O_2 concentrations on respiration in the 9.6-10.0 m soil depth of the cambisol and loess soils. Values are given as mean ± SEM (n=3).

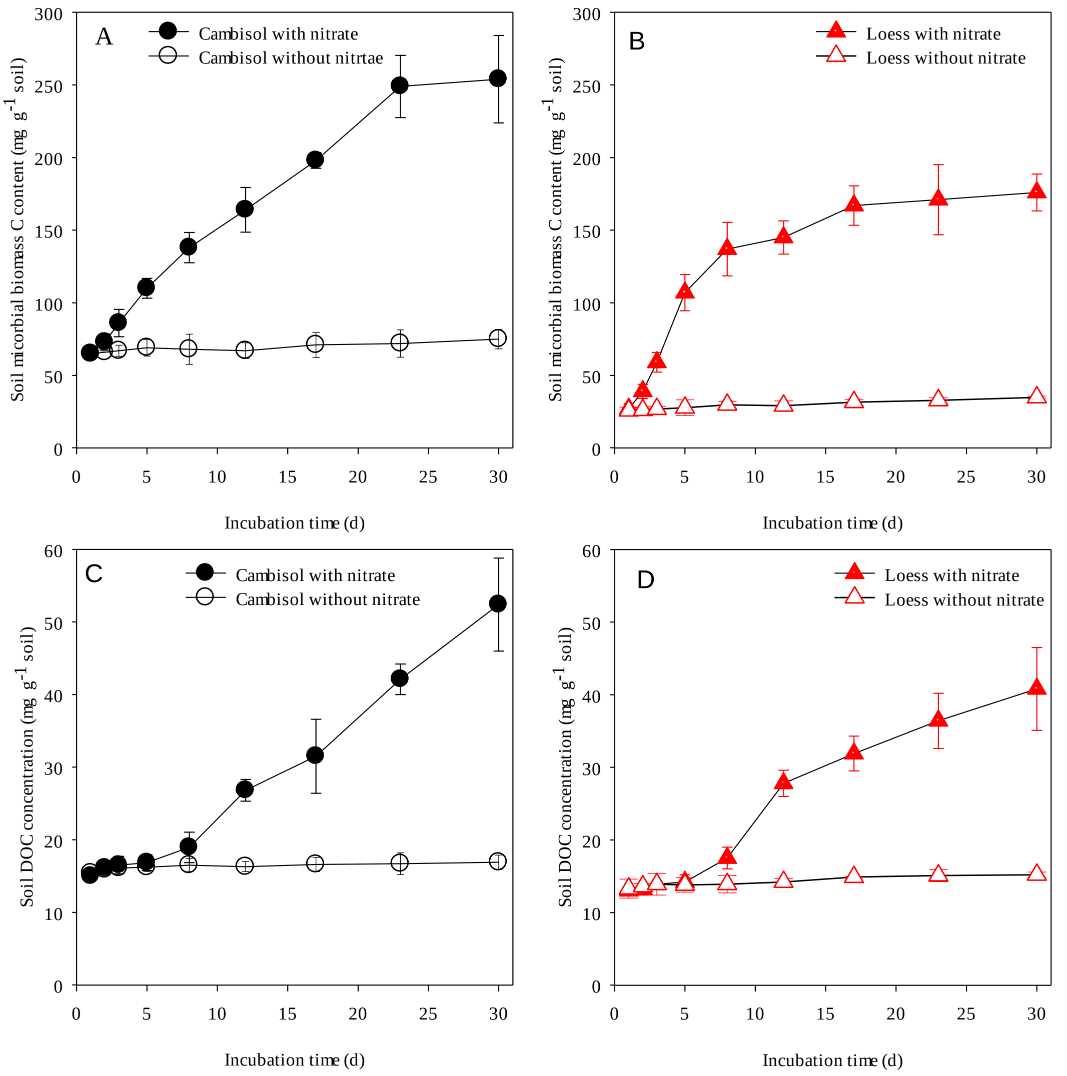

Fig. S5 Dynamics of microbial biomass C (A and B) and dissolved organic carbon (C and D) concentrations of the 9.6-10.0 m soil depth of the cambisol (A and C) and the loess soil (B and D) after nitrate amendment. Values are given as mean ± SEM (n=3).

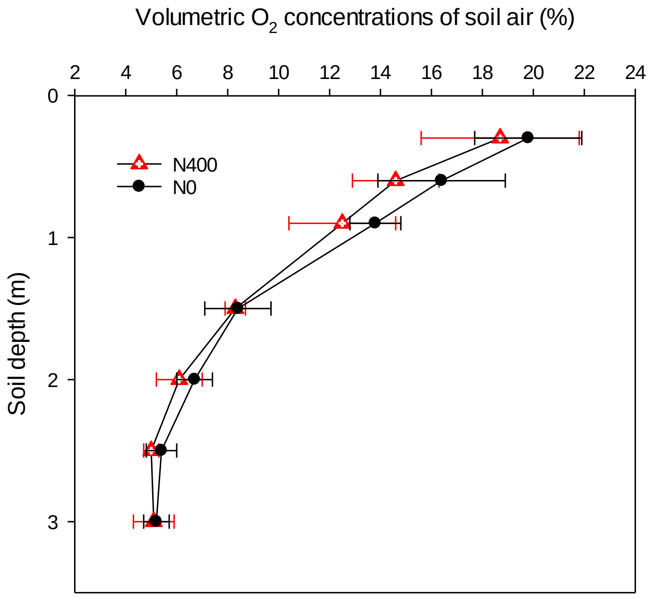

Fig. S6 Vertical distributions of the soil-air O_2 concentration in the 0-3 soil profile under the N0 and N400 treatments. Values are given as mean ± SEM (n=3).
